# Supplementary material for: Widespread genomic heterogeneity at the type II NAD(P)H dehydrogenase locus predisposes Cryptosporidium to clofazimine resistance
Source: bioRxiv. 2025 Oct 7:2025.10.07.680968. Preprint. [Version 1] doi: 10.1101/2025.10.07.680968 (PMC12632315; doi:10.1101/2025.10.07.680968)
Supplement: Supplement 1 [file NIHPP2025.10.07.680968v1-supplement-1.pdf]

bioRxiv preprint doi: https://doi.org/10.1101/2025.10.07.680968; this version posted October 7, 2025. The copyright holder for this preprint (which was not certified by peer review) is the author/funder, who has granted bioRxiv a license to display the preprint in perpetuity. It is made available under aCC-BY 4.0 International license.

Supplementary Information

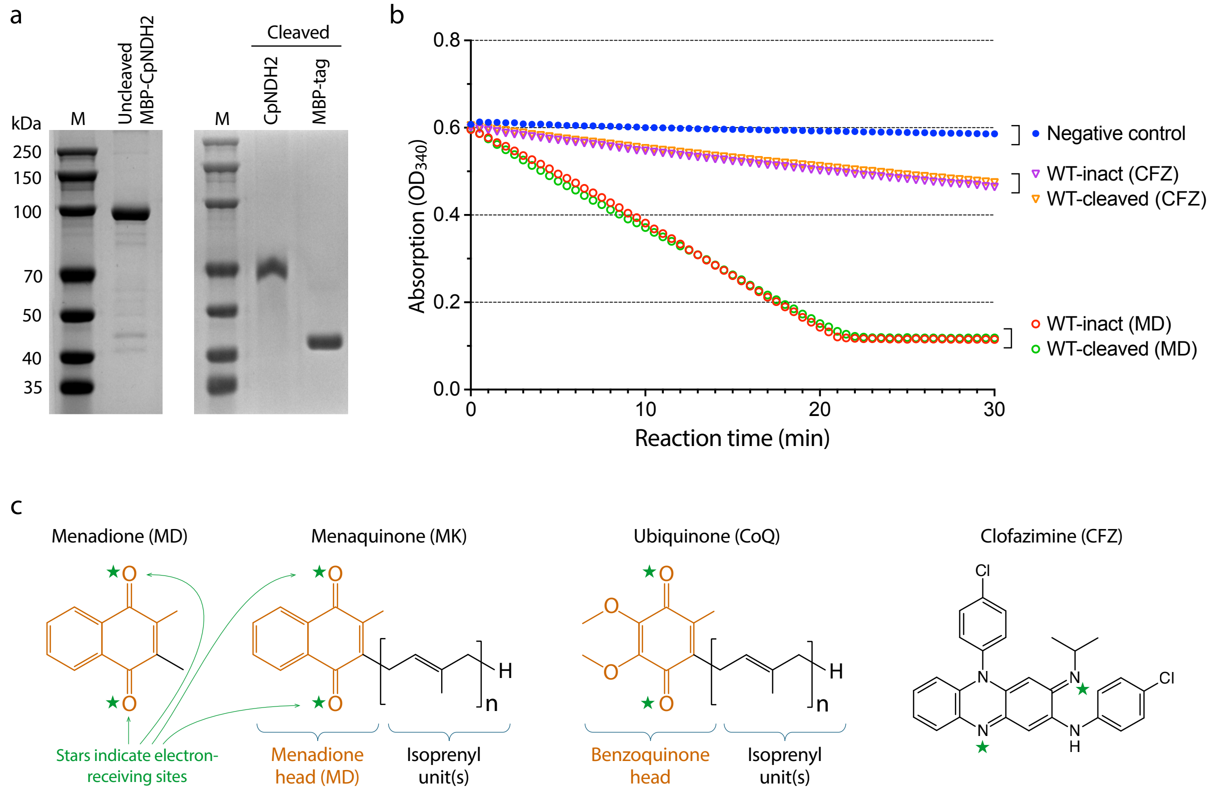

Supplementary Figure 1: Initial assessment of catalytic activity using purified recombinant wild-type CpNDH2 protein in intact and cleaved forms. (**a**) SDS-PAGE gels showing purified intact (uncleaved) and cleaved MBP-CpNDH2 protein. (**b**) Initial assessment of intact and cleaved CpNDH2 proteins in catalyzing the electron transfer from NADH to menadione (MD) and clofazimine (CFZ) using a spectrometeric assay.(**c**) Chemical structures of menadione, menaquinone, ubiquinone, and clofazimine. Green stars indicate atoms positioned to accept electrons during enzymatic reduction.

bioRxiv preprint doi: https://doi.org/10.1101/2025.10.07.680968; this version posted October 7, 2025. The copyright holder for this preprint (which was not certified by peer review) is the author/funder, who has granted bioRxiv a license to display the preprint in perpetuity. It is made available under aCC-BY 4.0 International license.

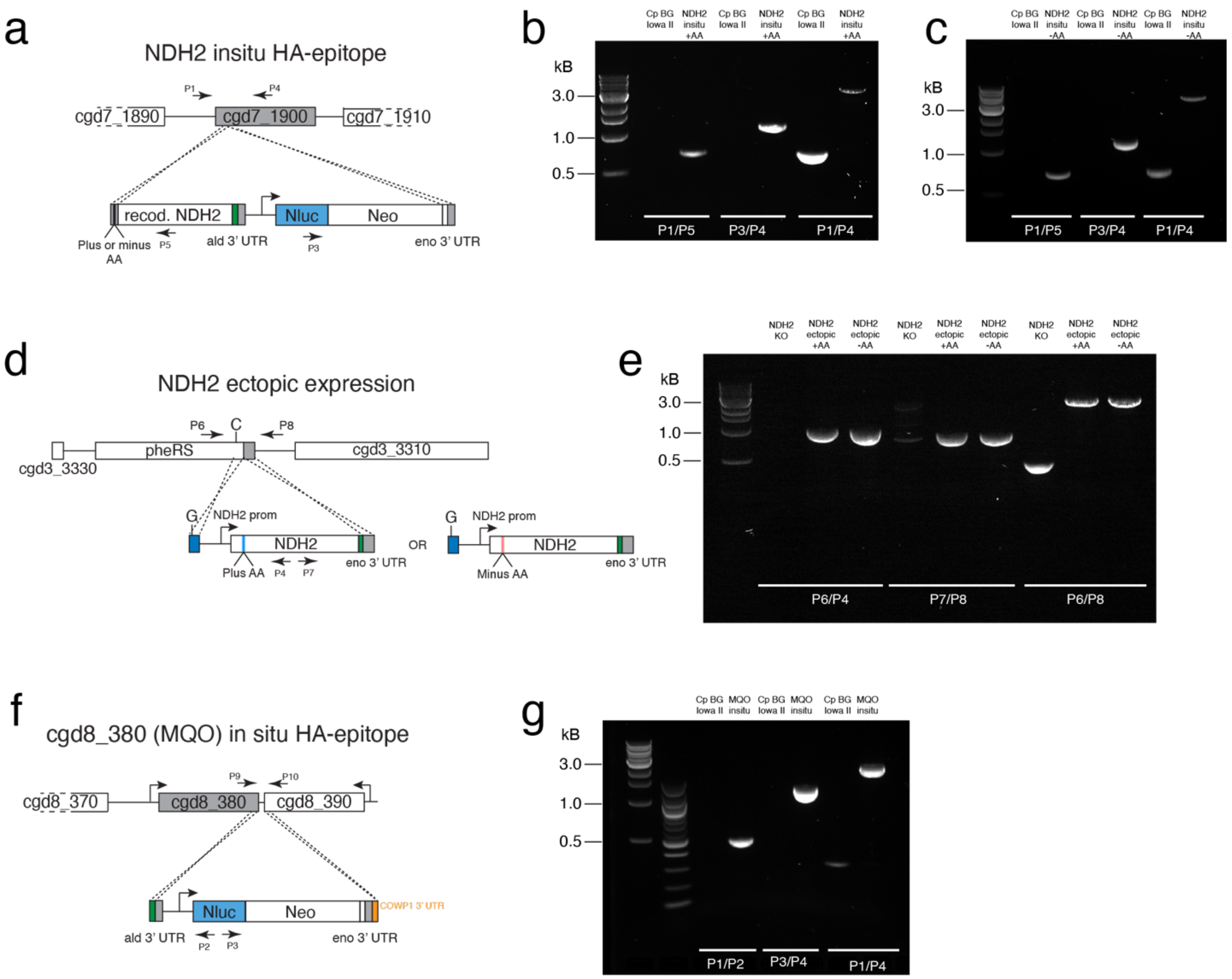

Supplementary Figure 2: Transgenic parasite strains constructed for this study.
(**a**) Map of NDH2 insitu HA epitope tagging strategy. Two strains were generated, either with or without INDEL (plus or minus AA). HA epitope = green. (**b**) Gel shows PCR mapping of the plus AA strain described in (a). (**c**) Gel shows PCR mapping of the minus AA strain described in (a). (**d**) Ectopic expression of NDH2-HA and NDH2-ΔAA-HA. HA epitope = green. (**e**) Gel shows PCR mapping of the strains described in (d). (d). (**f**) Map of MQO insitu HA epitope tagging strategy. HA epitope = green. (**g**) Gel shows PCR mapping of the MQO insitu HA epitope strain. Labelling of the gel refers to the amplicons shown in the maps (f). Labelling of all the gels refer to the amplicons shown in the respective maps.
